# Supplementary material for: Breakfast Consumption Suppresses Appetite but Does Not Increase Daily Energy Intake or Physical Activity Energy Expenditure When Compared with Breakfast Omission in Adolescent Girls Who Habitually Skip Breakfast: A 7-Day Randomised Crossover Trial
Source: Nutrients. 2021 Nov 26;13(12):4261. doi: 10.3390/nu13124261 (PMC8705113; doi:10.3390/nu13124261)
Supplement: Supplementary file 1 [file nutrients-13-04261-s001.zip › nutrients-1461558-supplementary.pdf]

Supplemental Table S1. Breakfast choices of adolescent girls who participated in randomised crossover trial comparing seven days of daily breakfast consumption (BC) with seven days of daily breakfast omission (BO)[1]

	Breakfast options	*n* for PA analyses sample	*n* for dietary analyses sample	*n* for VAS analyses sample
Breakfast cereal with semi-skimmed (1.8% fat) milk (compulsory)	Swiss Style Muesli (no added sugar)[2]	6	2	4
	Weetabix[3]	4	4	4
	All-Bran Original[4]	5	5	3
Raisins (optional)[2]		3	2	2
Beverage (compulsory)	Orange juice[2]	4	4	3
	Apple juice[2]	11	7	8

[1]Total *n*=15 for physical activity (PA) analyses; total *n*=11 for dietary analyses; total *n*=11 for visual analogue scale (VAS) analyses. BC was the consumption of a standardised breakfast with an energy content equating to 25% of individual resting metabolic rate before 09:00 for seven consecutive days; BO was the abstinence from all energy-providing nutrients until at least 10:30 for seven consecutive days.
[2]Tesco, UK.
[3]Weetabix Limited, UK.
[4]Kelloggs, UK.
